# Improved efficiency of coding systems with health information technology

**DOI:** 10.1038/s41598-021-89869-y

**Published:** 2021-05-13

**Authors:** Jinhyung Lee, Jae-Young Choi

**Affiliations:** 1 Department of Economics, and Samsung Advanced Institute for Health Sciences & Technology (SAIHST), Sungkyunkwan University College of Economics, Seoul, Republic of Korea; 2 Department of Business Administration, Hallym University College of Business, 1 Hallymdaehak-gil, Chuncheon, Gangwon-do 200-702 Republic of Korea

**Keywords:** Health care economics, Health policy, Health services, Public health

## Abstract

This study aimed to investigate the impact of health information technology (IT) on the Case Mix Index (CMI). This study was a retrospective cohort study using hospital financial data from the Office of Statewide Health Planning and Development (OSHPD) in California. A total of 309 unique hospitals were included in the study for 7 years, from 2009 to 2015, resulting in 2,135 hospital observations. The effects of health information technology (IT) on the Case Mix Index (CMI) was evaluated using dynamic panel data analysis to control endogeneity issues. This study found that more health IT adoption could lead to a lower CMI by improving coding systems. Policy makers, researchers, and healthcare providers must be cautious when interpreting the effect of health IT on the CMI. To encourage the adoption of health IT, the cost savings and reimbursement reductions resulting from health IT adoption should be compared. If any profit loss occurs (i.e., the cost savings is less than reimbursement reduction), more incentives should be provided to healthcare providers.

## Introduction

The Federal government of the United States (U.S.) has aggressively imposed use of the electronic health record (EHR) upon healthcare organizations by passing the American Recovery and Reinvestment Act of 2009. This act introduced both economic incentives and punitive actions. The use of EHRs can enhance coordinated care, reduce medical errors, and improve quality and patient safety^1–4^. Moreover, EHRs can enhance the accuracy of coding and the efficiency of reimbursement mechanisms^5^. EHRs may better document comorbid conditions to justify higher reimbursement rates from insurers to providers. Many insurers pay higher reimbursement rates for insured patients with a higher severity of illness (SOI) or with multiple comorbidities; thus, electronic documentation may improve charge capture^6^. Improved accuracy and efficiency of coding and billing might be correlated with the Case Mix Index (CMI), which is a measure to assess the clinical complexity, diversity, and the use of resources necessary to treat hospitalized patients.

It has been speculated that EHR systems might be misused either by manipulating data input or by processing data inappropriately to upcode claims^7^. Upcoding results from intentionally inflating the overall cost of care born by the insurer and society. Previous research on the relationship between the EHR adoption and coding behavior has shown mixed results^8–12^. Li^8^ and Ganju, et al.^9^ used the hospital-level CMI as the payment measure and found that health IT system adoption led to inflated reimbursement. Singh et al.^10^ showed EHRs facilitated the upcoding of evaluation and management codes in a large ophthalmologic practice. However, Adler-Milstein and Jha^11^ found no significant difference in hospital payments per discharge or in the change of a hospital’s CMI between IT adopters and non-adopters. They concluded that hospitals were not systematically using EHR systems to improve coding, thereby driving up costs. Park et al.^12^ examined the effect of health IT on the CMI and found that health IT was positively associated with the CMI, indicating that increased IT adoption could lead to a higher CMI or billing though diagnosis related group (DRG) up-coding. However, previous studies either specified limited functions such as EMR and computerized physician order entry (CPOE) among various health IT systems or used outdated datasets collected before the HITECH Act. The aim of this study is to examine the effect of health IT investment on the CMI by utilizing longitudinal data from the California Office of Statewide Health Planning and Development (OSHPD) from 2009 to 2015. We applied dynamic panel data analysis to control endogeneity issues.

## Methods

### Data

We used hospital financial data from the California government’s Office of Statewide Health Planning and Development (OSHPD) from 2009 to 2015. The data used was collected after the HITECH Act passed in 2009. The California hospital financial data provide characteristics about the organization in addition to financial information. These data have been used in some healthcare and economic studies^12,13^. The study sample was an unbalanced panel of hospitals with a total of 309 unique hospitals participated, for a total of 2,135 hospital year observations. Thus, the unit of analysis is hospital year.

### Dependent variable

For purposes of the study, the CMI was used as a dependent variable, which is the relative value assigned to the DRG of patients in a medical care environment. The CMI was applied to determine the allocation of resources to care for the patient groups^14^. To calculate the CMI, each patient treatment record was assigned to a Medicare Severity-DRG, based on patient characteristics. The Medicare Severity-DRG represents the consumption of national average hospital resources by patient group, relative to that of all patients^15^.

### Independent variable

Assets (non-IT) include Current Assets, Property, Plant and Equipment, Intangible Assets, Assets whose use is limited, and Other Assets. Labor (non-IT) is defined as the total conventional Salaries, Wages, Employee Benefits, and Professional Fees excluding any cost related to IT labor.

As a key explanatory variable, health IT expenditures are measured as a dollar amount and extracted from hospitals’ trial balance worksheets and supplemental information sheets. IT expenditures include IT capital-related costs (i.e., physical capital, purchased services, and lease/rental and other direct expenditures) as well as IT labor-related costs such as salaries and wages, employee benefits, and professional fees^14^.

In contrast with data sets used in previous studies, the OSHPD data did not provide the adoption status of each IT system. In order to examine the validity of continuous health IT measures, we examined the relationship between discrete measures of each health IT system (EMR, CPOE, PACS, patient billing, order entry, radiology information systems, clinical documentation, etc.) and health IT measures and found that the measure of IT system adoption was associated with IT cost. Thus, it showed IT investment could be the proxy of IT system adoption.

### Statistical analysis

We employed dynamic panel data (DPD) specifications to consistently estimate parameters under less restrictive assumptions than ordinary least square (OLS) and fixed effect (FE) panel data models. When serial correlation is detected, information exists in the error term instead of the estimated part of the model. In this case, the problem cannot be solved through estimation with robust standard errors, but must be investigated further by specifying and estimating a dynamic model. The DPD approach can simultaneously estimate the equation of interest using both levels and differences specifications where appropriate lags of the levels and differenced variables can be used as instruments. This simultaneous estimation strategy results in lower finite sample bias and increased precision. Thus, dynamic panel data analysis was adopted to examine the effect of IT on the CMI^15^. First we examined the model 1.

$$Model 1: {y}_{it}={\alpha }_{i}+ {\beta y}_{it-1}+{\theta }_{l}{l}_{it}+{\theta }_{k}{k}_{it}+\gamma I{T}_{it}+t+{\epsilon }_{it}$$

Here, *i* is hospital, *t* year. ${y}_{it}$ is the log of CMI, ${y}_{it-1}$ is the lagged term of the log of CMI, ${l}_{it}$ is the log of total labor, ${k}_{it}$ is the log of total capital, $I{T}_{it}$ is the log of information technology investment, t is the year effect, and ${\alpha }_{i}$ is the hospital fixed effect. In the equation above, ${\theta }_{l}$, ${\theta }_{k}$, and $\gamma$ are the input elasticities for each respective input.

Then, we examined the interaction effect of IT investment and Meaningful Use (MU) in model 2.

$$Model 2: {y}_{it}={\alpha }_{i}+ {\beta y}_{it-1}+{\theta }_{l}{l}_{it}+{\theta }_{k}{k}_{it}+{\gamma }_{1}{MU}_{it}+{\gamma }_{2}I{T}_{it}+{\gamma }_{3}I{T}_{it}{MU}_{it}+t+{\epsilon }_{it}$$

MU is defined as the stage 1, 2 and 3. MU stage 1 (MU1) was coded 1 before 2010 and 0 otherwise. Meaningful use stage 2 (MU2) was coded 1 between 2011 and 2012 and 0 otherwise. Meaningful use stage 3 (MU3) was coded 1 after 2012 and 0 otherwise. All analyses were conducted using Stata version 14 (Stata Corp College Station, Texas, USA) (https://www.stata.com/).

## Results

Tables 1 and 2 shows the descriptive statistics for the hospital financial variables and characteristics used. The average CMI was 1.26 and it increased by 1.7% annually over 7 years. The average labor cost was $196 million and it increased by 2.9% annually, and average assets were $301 million and increased by 6.7% annually in the same timeframe. Significantly, the IT investment almost doubled from $11.07 million to $20.7 million over the seven-year period. For hospital characteristics, average licensed bed was 246. The hospitals were more likely to be not-for-profit hospitals (61.1%) and less likely to be teaching hospitals (10.3%). Parameters of the DPD model are presented in Table 3. Serial correlation specification tests indicated that second differences removed the serial correlation and were used in the estimation. As the model is over-identified, the Hansen test for instrument validity was employed. The Hanson test p‐value was 0.41 indicating that the over-identification restrictions were not rejected.

**Table 1:** Descriptive statistics for hospital financial variables and characteristics (unit: hospital year).

Variables	Mean	S.D
CMI	1.26	0.35
Labor ($, million)	196.4	219.9
Assets ($, million)	301.3	448.1
IT cost ($, million)	15.4	32.8
Licensed beds	246	177
**Ownership (%)**		
Investor owned	488 (22.9%)	
Not-for-profit	1304 (61.1%)	
Public	342 (16.0%)	
Teaching status (%)	219 (10.3%)

**Table 2:** Descriptive Statistics for financial variables across year (unit: hospital year).

Year	CMI		Labor ($, million)		Assets ($, million)		IT Cost ($, million)	
	Mean	Std. dev	Mean	Std. dev	Mean	Std. dev	Mean	Std. dev
2009	1.2	0.34	174.34	193.62	237.61	333.30	11.07	21.48
2010	1.22	0.34	177.77	204.70	258.19	368.47	11.89	23.94
2011	1.23	0.32	193.64	210.17	280.43	399.80	14.09	31.88
2012	1.27	0.36	200.59	221.66	302.01	436.73	14.75	32.51
2013	1.29	0.36	207.95	230.70	327.73	476.39	16.01	33.19
2014	1.31	0.36	213.06	234.91	349.68	521.30	19.28	39.03
2015	1.33	0.37	206.18	237.26	348.91	542.99	20.7	41.26

**Table 3:** DPD regression results: a sample of 2,135 pooled observations representing 309 unique acute care hospitals in California operating between 2009 and 2015.

CMI	Model 1	Model 2
	Coef. (std)	Coef. (std)
L1.CMI	0.986***	0.986***
	(0.027)	(0.027)
Labor	− 0.012	− 0.013
	(0.009)	(0.009)
Asset	0.018**	0.019**
	(0.009)	(0.009)
**Meaningful use (MU)**		
Stage 1 (reference)		
Stage 2		− 0.030
		(0.040)
Stage 3		− 0.014
		(0.049)
IT cost	− 0.009*	− 0.01*
	(0.005)	(0.005)
IT cost * MU2		0.002
		(0.003)
IT cost * MU3		0.001
		(0.003)
Constant	0.056	0.074
	(0.072)	(0.082)

*p < 0.1, **p < 0.05; ***p < 0.01.

The DPD estimates indicate that IT was negatively associated with the CMI to a marginal extent (*p* < 0.1). For example, in Model 1, the CMI decreased by 0.09% when IT increased by 10%. While total assets were positively associated with the CMI, total labor was not significant. Additionally, The HITECH Act authorized up to $27 billion for an EMR incentive program over 10 years. The HITECH Act set meaningful use of interoperable EHR adoption in the health care system. In our sample, we examined the effect of meaningful use and the interaction of meaningful use and IT cost on the CMI in Model 2. However, we could not find the significant effect of meaningful use stage and interaction of meaningful use stage and IT cost. Nevertheless, the coefficients for IT costs and assets are similar to those of Model 1.

## Discussion

Recently, increasing concerns have emerged that the adoption of IT systems is likely to make it easier for providers to change patients' billing codes, and this could contribute to rising health expenditures and have an extensive impact on the healthcare industry. Further, it could negatively impact the data integrity and the quality of care if the coding system does not represent actual risk-adjusted quality measures. Overcoming methodological limitations in previous research, we found that health IT was inversely associated with patient severity of illness measured by the CMI, although the magnitude of the effect was relatively minor. Findings from this study are consistent with prior studies reporting lack of evidence of upcoding behavior arising from EHR adoption.

The modest inverse association of health IT and the CMI may imply that hospitals implementing EHR systems seem to selectively focus on certain complex and important conditions utilizing advanced technologies such as computer-assisted coding (CAC) tools combined with advanced natural language processing (NLP) technology to accurately document the severity of illness. The American Health Information Management Association (AHIMA) defines CAC as “the use of computer software that automatically generates a set of medical codes for review, validation, and use based upon clinical documentation”^16^. By automatically analyzing electronic documentation, CAC and NLP technology more accurately and completely identify major complications and comorbid conditions that impact the severity of illness than relying on manual coding. Previous studies reported that CAC tools with fully implemented EHR systems improve clinical coding accuracy due to greater consistency and improved capture in patient complexity level^17,18^. Meanwhile, hospitals seem to respond to Medicare and other federal and state policies. A recent study revealed that HITECH incentives were associated with a modest increase in the measured severity of illness determined by the number of condition categories from secondary discharge diagnosis codes^19^. Interestingly, the increase in the measured severity of illness associated incentives for health IT were concentrated among diagnoses targeted under the Hospital Readmissions Reduction Program (that is, acute myocardial infarction, heart failure, and pneumonia)^19^. This study reported an opposite pattern, decrease in the measured severity of illness, for untargeted conditions (all other conditions). While many insurers pay higher reimbursement rates for insured patients with higher severities or with multiple comorbidities, a higher CMI generally has a negative effect on hospital profitability^20^, implying that not all healthcare providers have incentive to maximize their CMI by intentionally and systematically upcoding claims. Consequently, the modest inverse association of health IT with the CMI we observed in the current study is less likely to support the evidence of fraudulent up-coding and more likely reflects better documentation.

It is worth mentioning that other factors were found to be associated with the CMI. Assets were found to have a positive effect on the CMI, implying that hospitals with larger assets might induce more severely ill patients. However, labor did not make any impact on the CMI. Additionally, MU 2 itself had no effect on the CMI. The Federal government set aside $27 billion for an incentive program that encourages hospitals and providers to adopt EMR. To receive these funds, providers must do more than simply purchase an EHR system. That is, they are required to show that they have achieved "meaningful use" of that system in terms of improving quality to receive the incentive. Thus, healthcare organizations needed to prepare for or begin IT investment before implementation of the HITECH Act. It could thus be interpreted that the HITECH Act itself could be a major factor in stimulating IT investment, although it is not directly associated with the CMI.

There is a limitation of the current study. The OSHPD database analyzed in the current work contains data only from California hospitals, so external validity to hospitals in other states in the United States and to hospitals in other countries is limited. Confirmation of these findings in other large administrative datasets in other geographic areas both within and beyond the United States is warranted. This is particularly important as our finding on association between CMI and health IT expenditure was marginally significant, and thus further studies with large dataset need to validate our findings.

## Conclusion

With the enactment of the HITECH Act, health IT investments have increased significantly. However, the impact of health IT on the CMI has not been well examined. We investigated the effect of IT on the CMI using hospital data from 2009 to 2015. The DPD regression results showed that health IT investment significantly and negatively affected the CMI.

This study has important policy implications. Healthcare providers should remember that reimbursement payments from insurers could be reduced by adopting health IT systems. However, they could save cost from health IT adoption through better coordination of care, reduction of medical errors, and adverse drug events (ADEs). On the other hand, the cost savings and reimbursement payment reduction from healthcare providers could reduce overall healthcare expenditures. Thus, health care policy makers may push healthcare organizations to adopt more health IT. This could result in a conflict of interest between healthcare providers and the healthcare policy makers regarding the adoption of health IT. Thus, to encourage the adoption of health IT, the cost savings and reimbursement reductions resulting from health IT adoption should be compared. If any profit loss occurs (i.e. the cost savings is less than reimbursement reduction), more incentives should be provided to healthcare providers.

## Data Availability

The datasets analyzed in the current study are available from the California government’s Office of Statewide Health Planning and Development (OSHPD).

## Code availability

Code is available from the authors upon reasonable request.
